# Perceived e-learning stress as an independent predictor of e-learning readiness: Results from a nationwide survey in Bangladesh

**DOI:** 10.1371/journal.pone.0259281

**Published:** 2021-10-28

**Authors:** Humayun Kabir, Sarker Mohammad Nasrullah, Md. Kamrul Hasan, Shakil Ahmed, Mohammad Delwer Hossain Hawlader, Dipak Kumar Mitra

**Affiliations:** 1 Department of Public Health, North South University, Dhaka, Bangladesh; 2 CRP Nursing College, Savar, Dhaka, Bangladesh; 3 IQARUS, Cox’s Bazar, Bangladesh; 4 Department of Biochemistry and Molecular Biology, Tejgaon College, National University, Bangladesh, Gazipur, Bangladesh; University of Hradec Kralove: Univerzita Hradec Kralove, CZECH REPUBLIC

## Abstract

**Background:**

E-learning is a relatively trending system of education that has been placed over conventional campus-based learning worldwide, especially since the emergence of the COVID-19 pandemic. This study aimed to assess e-learning readiness among university students of a developing country like Bangladesh and identify the independent predictors of e-learning readiness.

**Methods:**

From 26 December 2020 to 11 January 2021, a total of 1162 university students who had enrolled for e-learning completed a semi-structured questionnaire. Data were collected online via “Google Form” following the principles of snowball sampling through available social media platforms in Bangladesh. A multivariable linear regression model was fitted to investigate the association of e-learning readiness with perceived e-learning stress and other independent predictor variables.

**Results:**

A total of 1162 university students participated in this study. The results indicated that with the increase of students’ perceived e-learning stress score, the average e-learning readiness score was significantly decreased (β = -0.43, 95% CI: -0.66, -0.20). The students did not seem ready, and none of the e-learning readiness scale items reached the highest mean score (5.0). The age, gender, divisional residence, preference of students and their parents, devices used, and having any eye problems were significantly associated with the students’ e-learning readiness.

**Conclusion:**

During the prolonged period of the COVID-19 pandemic, e-learning implication strategies are needed to be assessed systematically with the level of readiness and its’ impacts among students for the continuation of sound e-learning systems. The study findings recommend evaluating the e-learning readiness of university students and the mental health outcomes during the COVID-19 catastrophe in Bangladesh.

## 1. Introduction

After more than a year since the emergence of SARS-CoV-2, it has now spread to nearly 200 countries around the globe with a staggering number of 120 million confirmed cases and 2.6 million deaths [1,2]. It has brought the world to its knees, imposing lockdowns, closure of academic institutions, inciting adverse psychological impacts, adopting the e-learning education system and work from home policy [3–5]. The world is relentlessly searching for new strategies to cope with the ‘new normal’ and resume daily work and business. E-learning is one such way of adapting to the current situation and continuing the education of billions of students confined in their homes worldwide [6,7].

Since the emergence of the worldwide web, its perimeter has expanded from instantaneous communication and data sharing to being an effective medium of online trade and management of official tasks, digital storage systems, and sources for all types of information imaginable [8]. In recent times, the tide has turned towards e-learning which provides flexible access to web-based teaching and learning tools. E-learning builds a platform for sustainable learning, which plays a pivotal role in improving the students’ skills and achievements through self-directed education [9]. Due to this reason, the world is leaning more and more towards e-learning systems, implementing them wherever the opportunity or necessity arises. Notably, in this pandemic, e-learning has opened a new window to continue the pedagogical activities and show light for future direction to cope with further catastrophe [10].

The concept of e-learning is defined as “the capabilities of the organization together with the educational authorities’ capabilities for effective and efficient application of electronic media” [11]. The idea has been in discussion in Turkey in recent years. Several e-learning programs have been under development, and experiments with different e-learning tools were underway to find the limitations and deficiencies of e-learning. Primarily the universities first began this slow adaptation with e-learning techniques. Hacettepe University in Ankara, Turkey, is one such institution [12,13]. The e-learning projects already in practice in Iran are expected to grow with increasing the students’ acceptance rate [14]. First-world countries like the USA, Canada, and others have already been offering e-learning based courses before the COVID-19 pandemic [15]. The unexpected emergence of the coronavirus has turned this progression towards e-learning into a quick transition to meet the demands of the situation that academics are facing worldwide.

Due to the pandemic, most of the educational institutions in Bangladesh were forced to shut down temporarily. In contrast, others had to take temporary measures to bring the students in touch with education again via online lectures and assignments. Albeit, the process might not be uniform, leaving a significant portion of the rural and sub-urban students out of the sphere. In addition, many teachers and students struggled due to the lack of experience and expertise in operating electronic devices and the application of e-learning tools [16,17]. Besides, the syllabi and curricula needed to be appropriately revised and adapted for e-learning [18]. The level of readiness, adequacy in the basic infrastructure, and proper use of technology were yet to evaluate regarding this matter. However, Ramírez-Correa et al. reported that the students’ perception of ease of use of technology was related to perceived enjoyment, external control, and adoption of e-learning [19].

Therefore, whether students were ready to adopt such technology or not was a significant concern. Moreover, the situation arose regarding students’ perceived stress experience due to this sudden shifting towards e-learning and added to the mental health problems that the coronavirus already created [20]. A study in Italy and Spain detected psychological issues among adolescent students owing to the pandemic [21]. Another study in Lebanon reported mild to moderate levels of stress was 12.7% of the subjects [22]. However, there was a knowledge gap between students’ e-learning readiness to adopt and the perceived stress level along the process.

The pandemic adversely affected people’s well-being worldwide and the students’ education at all levels. The academic curriculum of over 250 million students around the globe was disrupted due to this crisis [23]. In Bangladesh, over 5000 tertiary educational institutions enrolled around 4 million students were staying at their own homes trying to cope with the new learning strategies during the lockdown [24]. Notwithstanding, e-learning orientation in Bangladesh just after the early strike of the COVID-19 pandemic was in the question of readiness and their mental health burden [25]. Therefore, viable teaching strategies are a timely demand and minimize disease transmission for pedagogical continuity during or after the COVID-19 crisis [26].

Few studies assessed e-learning readiness and perceived stress among university students during the COVID-19 pandemic. To our best knowledge, this is the first study attempted to assess e-learning readiness and perceived stress among university students in Bangladesh. Our objective was to investigate the association of e-learning readiness with perceived e-learning stress and other predictor variables. This study will provide insights into the current situation of the e-learning education system in Bangladesh, leading to further researches and helping in future policy-making for the effective implementation of e-learning schemes during any catastrophe.

## 2. Methodology

### 2.1. Study settings

This study incorporated the cross-sectional design conducted from 26 December 2020 to 11 January 2021. The study respondents’ eligibility criteria included: (a) being under-graduate and graduate-level university students of Bangladesh; (b) enrolled in e-learning programs in their respective institutions (i.e., universities and graduate-level institutions) during the COVID-19 pandemic; (c) enrolled in the e-learning programs for at least 30 days; (d) willingly provided their online consent to participate.

### 2.2. Data collection procedure

A semi-structured questionnaire was used to collect data from the study respondents. To reduce and control the infection rate of SARS-CoV-2, all the educational institutions in Bangladesh have been closed since March 2020. Therefore, data were collected online with the principle of snowball sampling method via “Google Form” by using available social media platforms in Bangladesh, e.g., Facebook, WhatsApp, e-mail, etc. We selected three patterns for developing an online questionnaire: single option questions through multiple-choice, scale questions through linear scale options, and open questions through short answers. To measure e-learning stress and e-learning readiness, the Perceived Learning Stress Scale (PLSS) and e-learning readiness questionnaire were employed, respectively. In the study period, 1178 respondents provided their online consent to participate. During data cleaning, 16 respondents were excluded due to the incompleteness of the questionnaire. Finally, 1162 respondents’ information was included in the final analysis.

### 2.3. Selection of predictor variables

Apart from the primary predictor variable; perceived e-learning stress, to address the association with other predictor variables, we have collected information (sociodemographic and e-learning related variables) based on literature reviews, and experts’ opinions. The sociodemographic variables: e.g., age [27–31], gender [12,27–31], division [32], residence [32,33], monthly family income [34], degree [28,30,31], and parents’ highest education [35,36], and e-learning related variables: e.g., type of institution [27,31], students prefer e-learning [17,30,37], family prefers e-learning [36], device used for e-learning [29,38], having a single room [38], and having any eye problems [29] were included in this study.

### 2.4. Perceived e-learning readiness assessment

This study used an e-learning readiness questionnaire to assess e-learning readiness [12,39]. It consists of 39 items; scores range from 39 to 195. These items were responded to a five-point Likert scale 1 stands for “strongly disagree” and 5 for “strongly agree”. The questionnaire has five sub-domains of e-learning readiness, namely a) availability of technology (6 items), b) use of technology (11 items), c) self-confidence (12 items), d) acceptance (7 items), and e) training (3 items). Identification of expected e-learning readiness for each item of the questionnaire was defined as a mean score of 3.40 [12,39,40]. The reliability coefficient of the questionnaire, Cronbach alpha, was found 0.95, which seemed to be an excellent internal consistency. To check validity, we performed two approaches: (1) for content validity, the questionnaire was reviewed by experts along with that we took opinions from eminent educationists in Bangladesh; and (2) for construct validity, we conducted convergent validity by doing inter subscale correlation coefficient. We found that the subscales were moderately correlated.

### 2.5. Perceived e-learning stress assessment

The PLSS was used to measure perceived stress of e-learning (score range: 0–56) consisting of 14 items, and the responses were on a five-point Likert scale [41]. The scores were obtained by reverse scoring (e.g., 0 = 4, 1 = 3… & 4 = 0) of seven positive items (items 1, 2, 3, 8, 11, 12, and 14) and summing after all the 14 items. The higher score of PLSS indicated a higher level of perceived e-learning stress. On the other hand, for measuring perceived stress, Sheldon Cohen developed 14 items’ Perceived Stress Scale (PSS) that is general for measuring the degree of stress by asking participants about the feeling and thought of the last month [42]. Wherein Bangladesh, Nurul, and Mozumder validated the PSS among the adult population [43,44]. However, the PSS was used to measure learning stress previously among university students [45]. The PLSS is a slightly modified version of the PSS. Without changing the meaning of PSS queries, a slight phrasal modification was performed to reflect the context of e-learning in this study. In the PSS, an item reads for “In the last month, how often have you found that you could not cope with all the things that you had to do?” In our study, we added the word “of e-learning” in the first clause of the items. After the modification, reads for “In the last month of e-learning, how often have you found that you could not cope with all the things that you had to do?”. A similar modification and labeling of PSS as PLSS were performed by Lazarevic et al. for measuring the perceived stress of e-learning and face-to-face learning [41]. However, the PLSS showed adequate reliability in this study, where Cronbach alpha was estimated to be 0.86. For content validity of PLSS, we got the scale reviewed by eminent educationist in Bangladesh.

### 2.6. Data analysis

Descriptive statistics were performed for predictor variables (sociodemographic: e.g., age, gender, residence, division, monthly family income, degree, and parents’ highest education, and e-learning related information: e.g., type of institution, students prefer e-learning, family prefers e-learning, device used for e-learning, having a single room, and having any eye problems), e-learning readiness questionnaire, and PLSS. The perceived e-learning readiness questionnaire scores, subdomains of the perceived e-learning readiness questionnaire, and PLSS were expressed as mean, median, standard deviation, interquartile range (IQR), and score range. Cronbach alpha was estimated to measure scales’ reliability. Bivariate (unadjusted) linear regression models and a multivariable (adjusted) linear regression model were fitted to find the association of the perceived e-learning readiness with perceived e-learning stress and the other predictors. From the unadjusted linear regression model, we reported the unadjusted beta coefficient (***β***) with 95% confidence interval (95% CI), and from the adjusted linear regression model reported both the adjusted ***β*** and standardized ***β*** with 95% CI. Three hierarchical linear regression models were fitted to investigate the contributory role of the studied variables in students’ e-learning readiness. Multicollinearity of independent variables was tested by variance inflation factor (VIF). The highest VIF of the predictor variables was found 3.43, and the lowest was 1.09. The p-value <0.05 was considered as statistically significant at 95% confident interval. Data were analyzed by using statistical software STATA-14. Figures were prepared by using Prism 9.0.

### 2.7. Ethical issue

The Ethical Review Broad of the Faculty of Life Science, North South University, Bangladesh, approved this study. The reference number is 2021/OR-NSU/IRB/0601. The purpose of the study was explained on the first page of the survey questionnaire. Respondents were asked on the first question whether they were willing to participate, and those who selected ‘yes’ were considered to participate in the study.

## 3. Results

### 3.1. Characteristics of survey respondents

In this study, 1162 university students participated from all over Bangladesh. The background characteristics of the respondents are presented in **Table 1.** The majority of them were female (52.15%). More than two-thirds of them (69.10%) were in the B.Sc. program. The highest proportion was aged between 21 to 24 years (69.70%). More than 70% of the respondents were from the Dhaka division, and around 62% reside outside of Dhaka. A higher proportion of the respondents had a monthly family income of more than 30,000 BDT (354 USD) (51.26%). In terms of parents’ education level, more than one-third of the participants’ parents (43.63%) were at least graduates. The majority of the respondents (55.51%) were from a government institution. Although over half of the respondents (58.69%) reported having their preference for e-learning, notably, almost a similar proportion (58.78%) of the disapproval against e-learning came from the respondents’ families. Over three-fourths of the respondents continued e-learning programs using smartphones (75.99%). Most of the respondents (53.70%) had a single room for e-learning. The proportion of eye pathology (46.30%) (the doctors suggested that respondents not to stay for an extended period before any digital screen) was very high.

**Table 1:** Background characteristics of the study participants (n = 1162).

Background characteristics	Number	Percentage (%)/Mean (SD)
**Age (in years)**		
<21	193	16.61
21–22	428	36.83
23–24	382	32.87
>24	159	13.68
**Gender**		
Male	556	47.85
Female	606	52.15
**Division**		
Dhaka	818	70.40
Outside of Dhaka	344	29.60
**Residence**		
Urban	724	62.31
Rural	438	37.69
**Monthly family income**		
< 30,000 BDT	578	49.74
30,000 to <60,000 BDT	452	38.90
≥60,000 BDT	132	11.36
**Degree**		
Master’s	98	8.43
B.Sc.	803	69.10
Diploma	261	22.46
**Parents’ highest education**		
PhD	19	1.64
B.Sc.	243	20.91
Masters	245	21.08
H.S.C	233	20.05
S.S.C	216	18.59
Up to primary	206	17.73
**Type of institution**		
Government	645	55.51
Private	517	44.49
**Students prefer e-learning**		
No	480	41.31
Yes	682	58.69
**Family prefers e-learning**		
No	683	58.78
Yes	479	41.22
**Device used for e-learning**		
Desktop	93	8.00
Laptop	186	16.01
Hand set	883	75.99
**Having a single room**		
Yes	624	53.70
No	538	46.30
**Having any eye problems**		
Yes	538	46.30
No	624	53.70

### 3.2. Descriptive statistics of e-learning readiness questionnaires’ items

The mean score with SD of all 39 items is presented in **Table 2**. This study found none of the mean scores of the e-learning readiness questionnaire items to reach the maximum scores of 5. The mean scores differed between 2.14 (lowest for 1.39) and 4.22 (highest for 1.10), where the lowest score was related to the ‘training’ subdomain and the highest score was related to the ‘use of technology’ subdomain.

**Table 2:** Descriptive statistics of e-learning readiness questionnaires’ items (n = 1162).

Items	Mean	SD
**Availability of technology**		
1. The hardware facilities are enough.	3.16	1.23
2. The software facilities are enough.	3.20	1.22
3. The speed of the internet access is satisfactory.	2.80	1.32
4. The stability of the internet access is satisfactory.	2.87	1.33
5. I have access to computer whenever I need.	3.17	1.50
6. I can connect internet whenever I need.	3.14	1.44
**Use of technology**		
7. I use internet as information source.	4.08	1.07
8. I use e-mail as the main communication tool with my teachers and classmates.	3.33	1.33
9. I use office software (e.g. M.S. PowerPoint, Word, Excel).	3.32	1.43
10. I use social network sites (e.g. Facebook, Twitter).	4.22	0.99
11. I use specific software (e.g. SPSS).	2.54	1.40
12. I use instant messaging (e.g. Google Talk, Skype).	3.16	1.44
13. I use Web 2.0 tools (e.g. Blog, wiki) to share information.	2.48	1.36
14. I use file hosting services (e.g. Google Documents, Dropbox).	3.22	1.37
15. I use learning management systems (e.g. Blackboard, Moodle).	2.46	1.32
16. I use online forums and chat to communicate with my colleagues.	3.27	1.35
17. I use mobile technologies (e.g. Smartphone, Tablet) to connect internet.	4.20	1.02
**Self confidence**		
18. I have information about what e-learning is.	3.67	1.18
19. I have the skills to operate a computer.	3.81	1.21
20. I am able to use office software for content delivery and demonstration (e.g. M.S. Power Point, Word, Excel).	3.42	1.38
21. I am able to use web browsers (e.g. Internet Explorer, Google Chrome).	4.07	1.13
22. I am able to use search engines (e.g. Google, Yandex).	4.04	1.15
23. I can troubleshoot most problems associated with using a computer.	3.10	1.31
24. I can use digital file management tools (e.g. deleting or renaming a file on your computer).	3.57	1.32
25. I am able to do my homework by using electronic technology facilities.	3.65	1.22
26. I have enough time to prepare my homework by using electronic technology facilities.	3.47	1.22
27. I am able to use learning management systems (e.g. Blackboard, Moodle).	2.78	1.34
28. I believe that e-learning is easy to use.	3.49	1.22
29. I feel that I am ready for e-learning.	3.47	1.24
**Acceptance**		
30. I am keen to start e-learning.	3.58	1.27
31. I believe that e-learning can enhance the quality of education.	3.44	1.32
32. I believe that using e-learning can increase my productivity.	3.47	1.28
33. I believe that e-learning is more effectively than the traditional classroom-based approach.	3.03	1.38
34. I believe that e-learning enables learners and instructor to communicate and interact better with one another.	3.61	1.21
35. I believe that e-learning have benefits for education.	3.38	1.32
36. I support implementation of e-learning in my department.	3.43	1.28
**Training**		
37. I do not need training on e-learning.	2.35	1.26
38. My teachers do not need training on e-learning.	2.19	1.17
39. My classmates do not need training on e-learning.	2.14	1.16

Note: SD: Standard Deviation.

### 3.3. Descriptive statistics of e-learning readiness questionnaire and PLSS

This study found the availability of technology score range: 6–30, use of technology score range: 11–55, self-confidence score range: 12–60, acceptance score range: 7–35, and training score range: 3–15. The overall mean score of the questionnaire was found to be 127.78 (SD: 27.08), and the mean for all subdomains; availability of technology, use of technology, self-confidence, acceptance, and training was estimated to be 18.34 (SD: 6.17), 36.29 (SD: 8.72), 42.54 (SD: 10.62), 23.93 (SD: 7.60), and 6.69 (SD: 3.03), respectively. None of the five subdomains of the e-learning readiness questionnaire had shown Cronbach alpha of less than 0.80, which seems an excellent internal consistency. On the other hand, the mean score of PLSS was estimated to be 29.20 (SD: 5.87) in this study. All the mean scores score range, Cronbach alpha of the e-learning readiness questionnaire and PLSS are presented in **Table 3**.

**Table 3:** Descriptive statistics and Cronbach alpha of e-learning readiness questionnaire and PLSS (n = 1162).

Scales	Mean	Median	SD	IQR	Score range	Cronbach alpha
E-learning readiness	127.78	127	27.08	111–145	50–191	0.95
Availability of technology	18.34	18	6.17	14–23	6–30	0.84
Use of technology	36.29	35	8.72	31–42	11–55	0.84
Self confidence	42.54	43	10.62	36–50	12–60	0.91
Acceptance	23.93	24	7.60	19–29	7–35	0.93
Training	6.69	6	3.03	4–9	3–15	0.80
PLSS	29.20	29	5.87	26–32	8–50	0.86

Notes: SD: Standard Deviation, IQR: Inter Quartile Range.

### 3.4. Association of perceived e-learning readiness with perceived e-learning stress and other predictor variables

**Fig 1:**
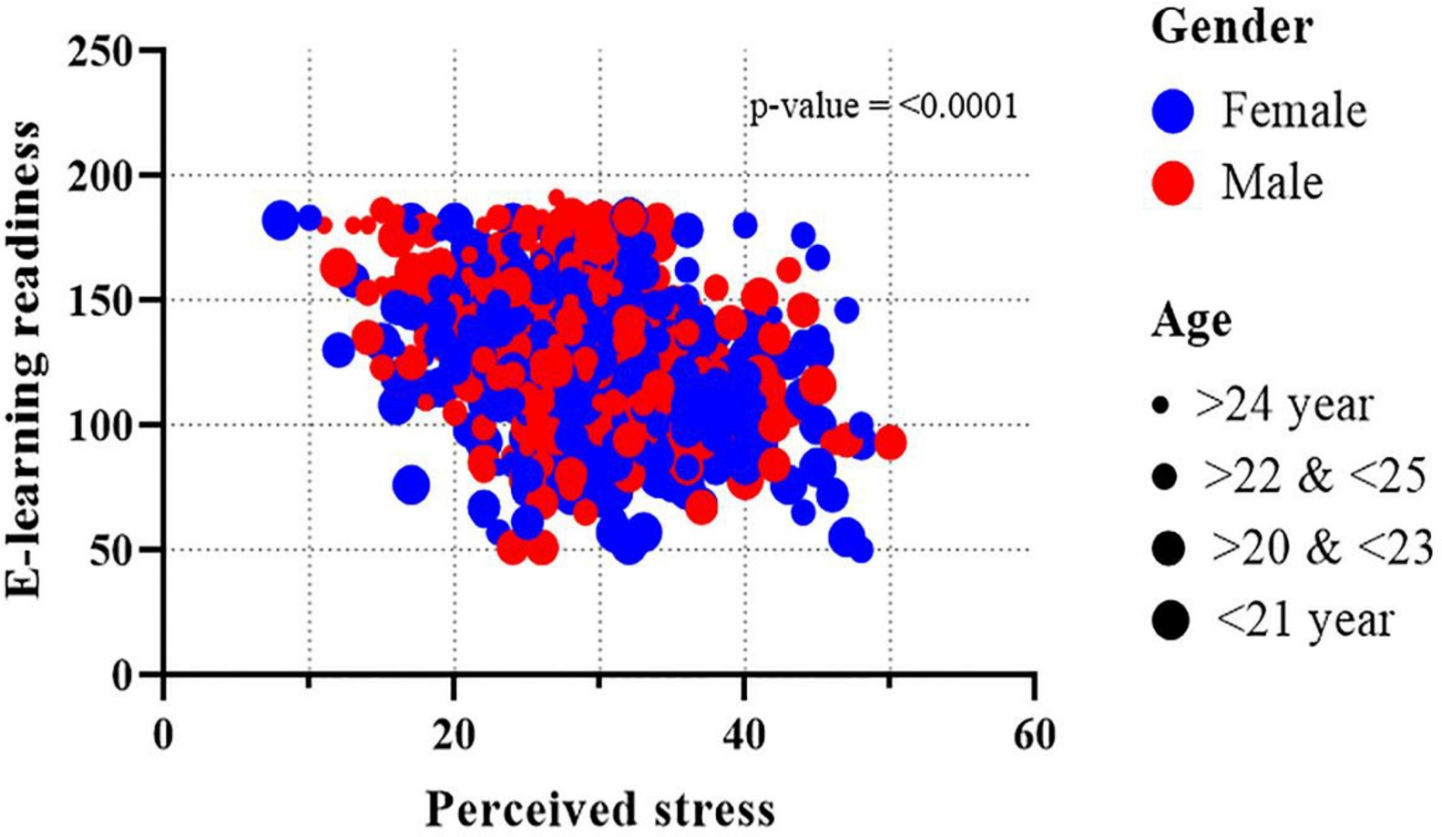
Association between perceived e-learning stress and e-learning readiness regarding gender and age.

**Fig 2:**
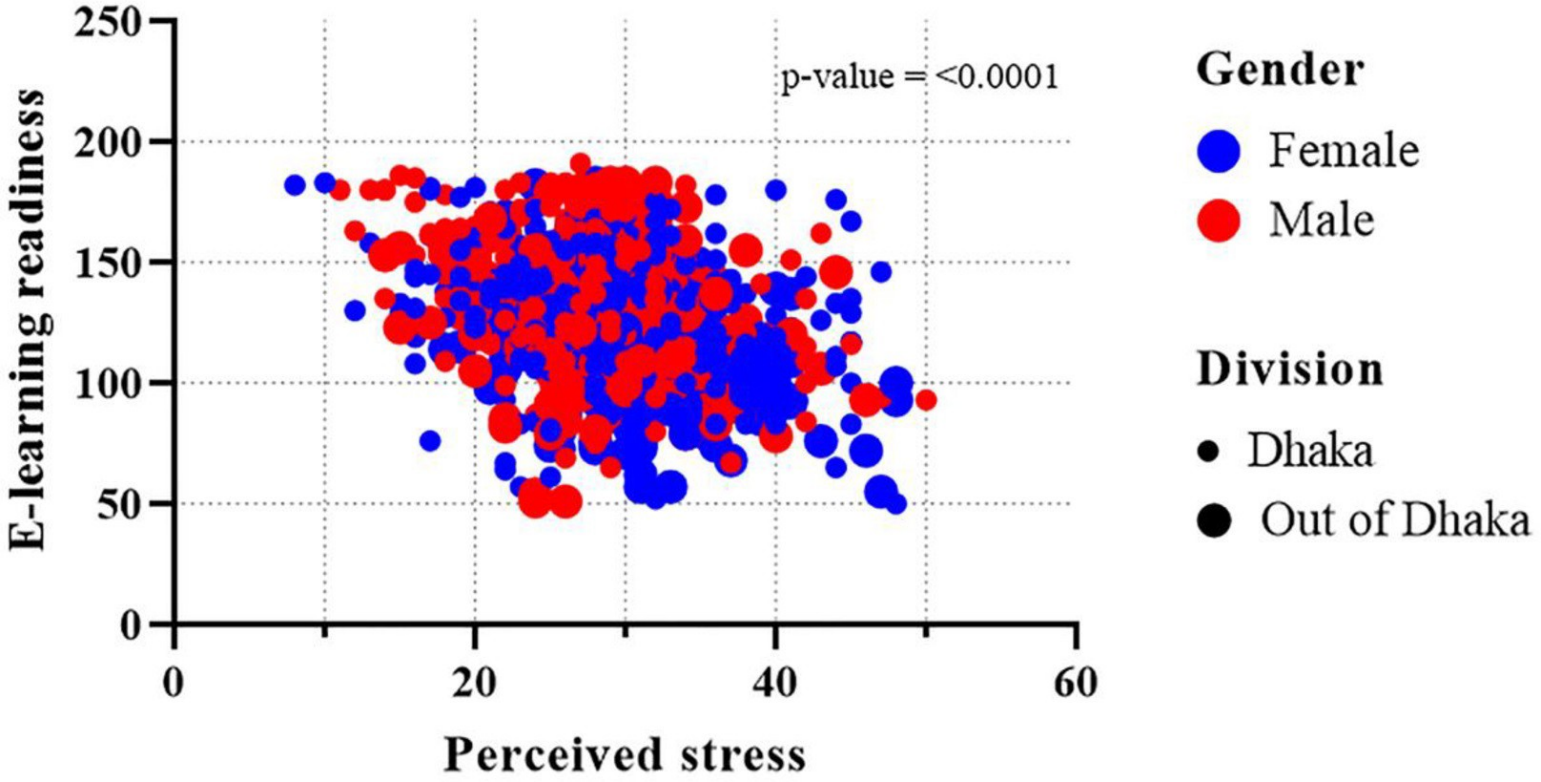
Association between perceived e-learning stress and e-learning readiness regarding gender and division.

**Table 4** shows the association of e-learning readiness with perceived stress and other e-learning readiness predictors. After adjustment, the results indicated that with the increasing trend of students’ perceived e-learning stress score, the average e-learning readiness score was significantly decreased (β = -0.43, 95% CI: -0.66, -0.20). In terms of age, the older age group i.e. 23–24 years (β = 5.14, 95% CI: 1.21, 9.07) had a significant difference in e-learning readiness compared to the younger group (<21 years: β = 5.25, 95% CI: 0.01, 10.48). Gender had a significant effect on e-learning readiness. Female students were not ready to e-learning (β = -4.85, 95% CI: -7.51, -2.18) as much as their male counterparts. Respondents’ residence played a significant role in e-learning readiness. Respondents from the Dhaka division (capital is in Dhaka) had higher e-learning readiness than those who were outside of Dhaka (β = -4.23, 95% CI: -7.24, -1.22). Besides, with the increasing pattern of the parent’s highest education, the average readiness score was significantly increased; parent’s highest educational status Ph.D. (β = 26.91, 95% CI: 16.31, 37.50), B.Sc. (β = 4.55, 95% CI: 0.14, 8.96), H.S.C. (β = 5.67, 95% CI: 1.50, 9.85) had higher average score compared to their less-educated compeer. However, the students who did not prefer e-learning got a significantly lower average score of readiness (β = -14.73, 95% CI: -18.03, -11.42). The same goes for the respondents’ families who preferred e-learning (β = 6.04, 95% CI: 2.78, 9.29) had higher average readiness scores. Whereas compared with the student’s enrollment to the e-learning program via the handset, the desktop and laptop enrolled students showed significantly higher average readiness score (β = 13.14, 95% CI: 8.24, 18.04) and (β = 10.79, 95% CI: 7.13, 14.45), respectively. Similarly, a significant difference was found for e-learning readiness in terms of having a single room compared to those who had to share their room (β = 5.76, 95% CI: 2.98, 8.54). The rest of the variables, i.e., residence, monthly family income, degree, types of institution, having any eye problems, showed no significant association with the e-learning readiness score after adjusting the multivariable linear regression model. However, a negative association (*p* = <0.001) was found between e-learning readiness and perceived e-learning stress concerning gender-age (**Fig 1**) and gender-division (**Fig 2**).

**Table 4:** Association of perceived e-learning readiness with perceived e-learning stress, and other predictor variables (n = 1,162).

Sample characteristics	Unadjusted *β* (95% CI)	*p*-value	Adjusted *β* (95% CI)	Standardized *β*	*p*-value
**Perceived stress**	-1.18 (-1.44, -0.93)	**<0.001**	-0.43 (-0.66, -0.20)	-0.09	**<0.001**
**Age**					
<21	Reference		Reference	Reference	
21–22	0.70 (-3.83, 5.22)	0.762	1.76 (-1.99, 5.51)	0.03	0.357
23–24	8.56 (3.96, 13.17)	**<0.001**	5.14 (1.21, 9.07)	0.09	**0.010**
>24	14.89 (9.30, 20.47)	**<0.001**	5.25 (0.01, 10.48)	0.07	0.09
**Gender**					
Male	Reference		Reference	Reference	
Female	-9.97 (-13.04, -6.90)	**<0.001**	-4.85 (-7.51, -2.18)	-0.09	**<0.001**
**Residence**					
Urban	Reference		Reference	Reference	
Rural	-11.83 (-14.98, -8.69)	**<0.001**	-2.07 (-4.95, 0.82)	-0.04	0.160
**Division**					
Dhaka	Reference		Reference	Reference	
Out of Dhaka	-10.17 (-13.54, -6.81)	**<0.001**	-4.23 (-7.24, -1.22)	-0.07	**0.006**
**Monthly family income**					
< 30,000 BDT	-14.80 (-19.84, -9.77)	**<0.001**	-2.54 (-7.12, 2.03)	-0.05	0.275
30,000 to <60,000 BDT	-6.77 (-11.94, -1.61)	**0.010**	-1.94 (-6.25, 2.36)	-0.04	0.376
≥60,000 BDT	Reference		Reference	Reference	
**Degree**					
Master’s	17.78 (11.57, 23.99)	**<0.001**	2.14 (-4.21, 8.49)	0.02	0.509
B.Sc.	6.93 (3.19, 10.67)	**<0.001**	-2.06 (-5.77, 1.67)	-0.04	0.279
Diploma	Reference		Reference	Reference	
**Parent’s highest education**					
PhD	47.45 (35.15, 59.76)	**<0.001**	26.91 (16.31, 37.50)	0.13	**<0.001**
B.Sc.	13.21 (8.35, 18.07)	**<0.001**	4.55 (0.14, 8.96)	0.07	**0.043**
Masters	10.19 (5.33, 15.04)	**<0.001**	2.62 (-1.65, 6.89)	0.04	0.229
H.S.C	9.15 (4.24, 14.06)	**<0.001**	5.67 (1.50, 9.85)	0.08	**0.008**
S.S.C	1.33 (-3.79, 6.30)	0.610	1.06 (-3.08, 5.19)	0.02	0.617
Up to primary	Reference		Reference	Reference	
**Type of institution**					
Government	2.49 (-0.65, 5.62)	0.119	-0.63 (-3.49, 2.22)	-0.01	0.664
Private	Reference		Reference	Reference	
**Students prefer e-learning**					
No	-25.55 (-28.35, -22.74)	**<0.001**	-14.73 (-18.03, -11.42)	-0.27	**<0.001**
Yes	Reference		Reference	Reference	
**Family prefers e-learning**					
No	Reference		Reference	Reference	
Yes	21.55 (18.64, 24.47)	**<0.001**	6.04 (2.78, 9.29)	0.11	**<0.001**
**Device used for e-learning**					
Desktop	27.60 (22.18, 33.01)	**<0.001**	13.14 (8.24, 18.04)	0.13	**<0.001**
Laptop	19.40 (15.39, 23.41)	**<0.001**	10.79 (7.13, 14.45)	0.15	**<0.001**
Hand set	Reference		Reference	Reference	
**Having a single room**					
Yes	17.97 (15.02, 20.93)	**<0.001**	5.76 (2.98, 8.54)	0.11	**<0.001**
No	Reference		Reference	Reference	
**Having an eye problems**					
Yes	-5.86 (-8.97, -2.75)	**<0.001**	-2.40 (-4.98, 0.18)	-0.04	0.069
No	Reference		Reference	Reference	

Notes: *β*: Coefficient, CI: Confidence Interval.

### 3.5. Predictive models of the students’ e-learning readiness

In **Table 5,** hierarchical linear regression models are presented. Three models were fitted in analysis to estimate the contributory role of the studied variables to predict students’ e-learning readiness. In model-1, we included only the perceived e-learning stress score, model-2 considered the sociodemographic characteristics, and in model-3, e-learning related information was added. All the models were significantly associated with students’ e-learning readiness. About 6% variance of e-learning readiness was explained by perceived e-learning stress, and after adding sociodemographic characteristics in model-2, 19% variance was explained. In the final model-3, 37.2% total variance of e-learning readiness was explained just after adding e-learning related information along with perceived e-learning stress and sociodemographic characteristics.

**Table 5:** Hierarchical linear regression models to predictor students’ e-learning readiness (n = 1162).

Predictors	Model-1 (F = 82.34, R² = 0.066, f, adjusted R² = 0.066, p < 0.001)			Model-2 (F = 33.88, R² = 0.190, adjusted R² = 0.185, p < 0.001)			Model-3 (F = 48.49, R² = 0.372, adjusted R² = 0.3641, p < 0.001)		
	***β***	SE	95% CI	*β*	SE	95% CI	*β*	SE	95% CI
Constant	162.29	3.88	154.68,169.90	143.21	7.21	129.07, 157.35	113.52	8.87	96.11, 130.92
Perceived stress	-1.18	0.13	-1.44, -0.93	-1.03	0.12	-1.27, -0.79	-0.41	0.12	-0.64, -0.18
Age				2.40	0.84	0.76, 4.04	2.08	0.75	0.62, 3.54
Gender^a^				8.64	1.50	5.71, 11.58	4.84	1.36	2.17, 7.51
Residence^b^				4.96	1.65	1.73, 8.20	2.10	1.48	-0.80, 4.99
Division^c^				-5.87	1.72	-9.26, -2.49	-4.40	1.53	-7.41, -1.40
Monthly family income^d^				3.18	1.19	0.85, 5.50	1.05	1.07	-1.05, 3.14
Degree^e^				1.50	1.17	-0.79, 3.79	2.14	1.06	0.06, 4.22
Parents’ highest education^f^				-3.03	0.55	-4.11, -1.96	-1.51	0.50	-2.50, -0.53
Type of institution^g^							0.28	1.34	-2.36, 2.92
Students prefer e-learning^h^							15.02	1.69	11.70, 18.34
Family prefers e-learning^i^							5.79	1.67	2.52, 9.06
Device used for e-learning^j^							-7.93	1.13	-10.15, -5.71
Having a single room^k^							6.30	1.41	3.52, 9.07
Having any eye problems^l^							-2.28	1.32	-4.87, 0.30

^a^1 = Female, 2 = Male.

^b^1 = Urban, 2 = Rural.

^c^1 = Dhaka, 2 = Outside of Dhaka.

^d^1 = < 30,000 BDT, 2 = 30,000 to < 60,000 BDT, 3 = ≥ 60,000 BDT.

^e^1 = Masters, 2 = B.Sc., 3 = Diploma.

^f^1 = PhD, 2 = B.Sc., 3 = Masters, 4 = H.S.C, 5 = S.S.C, 6 = Up to primary.

^g^1 = government, 2 = Private.

^J^1 = Desktop, 2 = Laptop 3 = Hand set, ^h, i, k, l^1 = Yes, 2 = No.

## 4. Discussion

This study investigated e-learning readiness and perceived stress during the COVID-19 catastrophe among university students of a developing country like Bangladesh. The participants were both undergraduate and graduate-level university students who were included from all across the country. This study revealed a significant association between perceived stress and e-learning readiness. Age, gender, parents’ highest education level, preference of e-learning by learners and their parents, the device they used, and having a single room for e-learning were also evidently associated with the e-learning readiness. To our best knowledge, this study is the first of its kind during the COVID-19 pandemic in a developing country like Bangladesh that was carried out to provide an insight into students’ e-learning readiness and perceived e-learning stress.

The study revealed that none of the items reached the highest mean score (5.0) of the e-learning readiness questionnaire. The lowest mean score (2.14) was observed among the students regarding ‘classmates need not training’ for e-learning. The mean scores of teachers’ (2.19) and students’ (2.35) training items were also found low. Similarly, Farhana et al. reported that the lack of training of the Bangladeshi teachers for e-learning profoundly challenged the implication of e-learning during the COVID-19 outbreak [9]. The finding of this study indicated that the students and teachers lacked training, thus providing it may upheave students toward readiness. However, Ngampornchai et al. reported that trained students were perceived to have e-learning readiness more than non-trained students [37].

In terms of perceived e-learning stress, this study found a significantly negative association with e-learning readiness. In the adjusted model, age, gender, divisional residence, parents’ highest education, students and their family preference, device used, and having a single room were significantly associated with e-learning readiness. Similarly, prior studies investigated students’ perceived stress and e-learning readiness during and before the COVID-19 pandemic [37,46,47]. Keller et al. reported that previous experience with technologies is an influential predictor of students’ positive attitudes towards e-learning implementation strategies [48]. Essentially, Händel et al. observed that technologically equipped students had more readiness and significantly less stress regarding e-learning [49].

Similar to our findings, Heo et al. reported that stress is a significant predictor of e-learning readiness [50]. However, Ateeq et al. revealed that the source of stress among students could be a combination of academic, social, and financial issues during the COVID-19 catastrophe [46]. Thus, it can be stated that increased perceived e-learning stress might pose decreased or poor trend of e-learning readiness among the students.

The senior students were observed to be more ready for e-learning in this study. Besides, a study found that self-efficacy of computers, the internet, and online communication is significantly higher among senior students [51]. Albeit, Heo et al., and Emami H. found no significant effect of seniority on e-learning readiness [50,52]. The male students were found to have more e-learning readiness in this study. Similarly, an online survey in Germany reported that female students were less ready for e-learning than male students [49]. However, regarding e-learning readiness, Emami H. found no significant difference between males and females [52]. Therefore, perhaps e-learning readiness may be associated with seniority and gender.

Elnakeeb et al. reported that urban students had more computer and internet-based self-efficiency than rural [51]. A study in India found residence in rural areas to hinder the implementation of e-learning in students regarding the availability of digital technology [53]. In the adjusted model, our study found urbanity as an insignificant predictor of students’ e-learning readiness. This variation might be explained as the students’ backed returned to their rural home before the COVID-19 lockdown [54]. On the other hand, the students outside of the Dhaka division were revealed significantly less ready for e-learning. This variation could be explained by the poor e-learning infrastructure and lack of availability of technology or poor internet access to the Dhaka division versus outside of Dhaka. Whereby, the internet services in Bangladesh are unsatisfactory in terms of both speed and stability [55]. Therefore, any distraction or constraint of technology might be an issue for students’ e-learning experience [53]. Henceforth, for successful e-learning strategies, stable internet connectivity requirements should be fulfilled at an optimal level [53]. Additionally, Dhaka is a highly infrastructural division, where the capital of Bangladesh belongs [56].

The family income was found to be an insignificant predictor of e-learning readiness in the adjusted model. However, Barteit et al. reported that lower socioeconomic status predicted e-learning readiness [57]. A review study found the economic status as a challenge of e-learning implementation strategies [10]. We did not find family income as a significant predictor of e-learning readiness, it might be that some of the participants were shy to express the family income, or perhaps they did not want to disclose their exact family income. On the other hand, our study found parents’ educational status as a significant predictor of e-learning readiness. To succeed in an e-learning program, the teachers sometimes need to communicate and coordinate with parents, whereby the teacher and parents may be affected by the reduction of working hours [58]. Therefore, in terms of parents’ education, our study finding concludes that highly educated parents might have the potential to make students profoundly ready for receiving e-learning.

In this study, e-learning readiness was observed to be significantly higher in those who prefer e-learning. In line with our finding, Muthuprasad et al. found that the availability of the internet and proper skills of technology attributed to e-learning preference among students [53]. However, family preference was also found to be significant for improving e-learning readiness. According to Kong et al., parents with a positive perception of e-learning play a supportive role and understand the pupils’ demands [35]. Our finding concludes that the parents’ preference is required to provide all facilities to attend e-learning. Thus, both the participants and family preference seemed to be crucial for e-learning readiness.

Increased screen time was linked to digital eye strain in previous studies [29,59]. However, in the study at hand, non-significant correlations were found between vision problems and e-learning readiness. Still, there might be a trend towards a relationship between vision and e-learning readiness. On the other hand, students’ enrolled with desktop and laptop were found to be more ready than those enrolled with the handset (mobile, tablet) significantly. This difference could arise from those accustomed to using computers which could have a better grip on e-learning related applications and online management tools, as using mobile phones instead restricts the process due to a few limitations [60]. However, according to BTRC (Bangladesh Telecommunication Regulatory Commission), 94% of Bangladeshi people use the internet on mobile phones [61].

Lastly, having a single or separate room for e-learning was a significant predictor of e-learning readiness in this study. This variation could be explained by the fact that having a separate room for studying away from distractions could help to be focused on the tasks. This finding is supported by a study conducted in India showing improvement in students’ learning status when provided with private spaces and a calm environment [62]. A calm and quiet environment might be paving the way for vigilant e-learning readiness.

## 5. Strength and limitation

To our best knowledge, this is the first study in Bangladesh, which tried to identify predictors of e-learning readiness from the view of perceived e-learning stress. The authors followed a study of Lazarevic et al. that concentrated on PLSS, a modified version of PSS [41]. Lazarevic et al. reported the reliability in their study (Cronbach alpha: 0.83). Hence, we performed content validity and reliability. A few factors might be surfaced out through that process as if workload might play a role on PLSS. Moreover, as this study took place amidst the COVID-19 pandemic, the pandemic itself could be a stressor, which might interfere with e-learning readiness. Different reasons for chronic stress and stress before COVID-19 were not assessed in this study.

As it was based on a cross-sectional study design, our study could not explain causality between the variables. In addition to that, selection bias might be occurred due to the snowball sampling method. Henceforth, the authors recommend performing further research to mark the predictors of e-learning readiness more broadly, encompassing different types of stress, not only concentrating on specific conditions like the COVID-19 pandemic for a robust, sustainable, and equitable education system. The subjects of this study were recruited from different universities across the country; therefore, the study findings could be generalized in Bangladesh and other lower-middle-income countries for the population with corresponding academic and demographic characteristics.

## 6. Conclusion

This research can cogent that e-learning readiness had a significant association with perceived e-learning stress among university students. Moreover, several variables such as age, gender, parents’ highest education level, preference for e-learning by learners and their parents, devices they used for online classes, and having a single room were the potential predictors of e-learning readiness.

The study findings could be the first step towards a balanced pedagogical system for emergencies like the COVID-19 pandemic and at regular times. Significantly, the responses in the five different domains of e-learning provide crucial insight into the present conditions of e-learning practices in the country. For example, “availability of technology” and “use of technology” can come in handy to boost up the “confidence” and encourage “acceptance” further. Finally, yet importantly, the training of respected teachers can be pivotal in strengthening the backbone of e-learning. As the world progresses rapidly in technological advancement, students residing in the remotest part of the country will get similar opportunities to receive quality education through distance education. In addition, the results of this study tell us principally about university students’ readiness and discuss its’ importance as a catalyst for a sound e-learning system. Based on the research findings, the university authority, teachers, and policymakers should pay more attention to the e-learning readiness and the students’ mental health outcomes for the sustained continuation of e-learning, especially during this global crisis and thus managing the catastrophe pragmatically.

## Supporting information

S1 File (DOCX)
