# Supplementary material for: Perceived e-learning stress as an independent predictor of e-learning readiness: Results from a nationwide survey in Bangladesh
Source: PLoS One. 2021 Oct 28;16(10):e0259281. doi: 10.1371/journal.pone.0259281 (PMC8553166; doi:10.1371/journal.pone.0259281)
Supplement: S1 File — (DOCX) [file pone.0259281.s001.docx]

**Most acknowledgeable Research Assistant names**

| **Name** | **Affiliation** |
| --- | --- |
| 1. Shimpi Akter | Japan Bangladesh Friendship Nursing College, Dhaka |
| 1. Rawshan Ara | Prime College of Nursing College, Dhaka |
| 1. Tasnova Ojifa | STS Nursing College, Dhaka |
| 1. Anjan Roy Raj | Jashore University of Science & Technology, Jashore |
| 1. Shyjuddin Khan 2. Bipasha Akter Shopna 3. Shilpe Akter 4. Md. Abdul Quam 5. Md Ikbal Hossain 6. Sharika Tahsin 7. Sohel Mahmud 8. Afia Ayub 9. Easin Arafat 10. Nusrat Bhuiyan 11. Iffat Ara | 250 Beded General Hospital, Brahmanbaria  CRP Nursing College, Savar  Dynamic Nursing College, Dhaka  AM Nursing College, Maulvi Bazar  Begum Rabeya Khatun Chowdhury Nursing College, Sylhet  Tejgaon College, National University of Bangladesh, Gazipur- 1704,  Bangladesh Tejgaon College, National University of Bangladesh, Gazipur- 1704, Bangladesh  Tejgaon College, National University of Bangladesh, Gazipur- 1704, Bangladesh  Tejgaon College, National University of Bangladesh, Gazipur- 1704, Bangladesh  Tejgaon College, National University of Bangladesh, Gazipur- 1704, Bangladesh  Tejgaon College, National University of Bangladesh, Gazipur- 1704, Bangladesh |
